# Resumption of production operations during the silent disposal period of African swine fever outbreaks: a case study

**DOI:** 10.1186/s40813-023-00338-6

**Published:** 2023-10-10

**Authors:** Zhiqiang Hu, Qingyuan Liu, Xiaogang Tian, Lulu Li, Weisheng Wu, Wenchao Gao, Xiaowen Li

**Affiliations:** 1 Shandong Engineering Laboratory of Pig and Poultry Healthy Breeding and Disease Diagnosis Technology, Xiajin New Hope Liuhe Agriculture and Animal Husbandry Co., Ltd, Xiajin Economic Development Zone, Qingwo Venture Park, Dezhou, 253200 Shandong Province P. R. China; 2 Key Laboratory of Feed and Livestock and Poultry Products Quality & Safety Control, Ministry of Agriculture and Rural Affairs, New Hope Liuhe Co., Ltd, 316 Jinshi Road, Chengdu, 610100 Sichuan P. R. China; 3 Shandong New Hope Liuhe Co.,Ltd, No. 592-26 Jiushui East Road Laoshan District, Qingdao, 266100 Shandong P. R. China; 4 Shandong New Hope Liuhe Agriculture and Animal Husbandry Technology Co., Ltd (NHLH Academy of Swine Research), 6596 Dongfanghong East Road Yuanqiao Town, Dezhou, 253000 Shandong People’s Republic of China; 5 China Agriculture Research System-Yangling Comprehensive test Station, Intersection of Changqing Road and Park Road 1, Yangling District, Xianyang, P. R. China

**Keywords:** African swine fever, Non-productive days, Retention rate, Economic loss, Cost implication, Epidemic disposal, Positive detection

## Abstract

**Background:**

Batch production, a widely implemented production model in large-scale pig farms, was characterized by its long-term duration, cost-effectiveness, and efficiency. Nevertheless, the recent occurrence of African swine fever (ASF) outbreaks in China has necessitated the implementation of discreet mating operations within this model, leading to disruptions in production cycles and substantial indirect losses.

**Case presentation:**

This study implemented a novel operational procedure, which involved the division of risk areas for zone management and allowed mating operations, in 12 farms experiencing ASF outbreaks. Another 12 farms were used as a control group, employing the old procedure. Subsequently, the prognoses of both the old and new procedures were calculated and analyzed. The findings indicate that the new method resulted in an enhanced retention rate and reduced non-productive days (NPD), without impacting the positive detection rate and disposal time. Consequently, this approach significantly mitigated economic losses (p < 0.05).

**Conclusion:**

The efficacy of the novel procedure in mitigating the indirect economic losses stemming from ASF outbreaks, through the reduction of NPD while maintaining retention rates and disposition days, has been substantiated. This methodology has demonstrated feasibility in extensive pig farming operations and exhibits promise for broader application.

## Background

The concept of batch production was initially introduced in 1935 and has since been implemented in extensive pig farming operations, demonstrating its long-term viability, cost-effectiveness, and efficiency. The merits of batch production are manifold. Firstly, it enables precise forecasting of production plans in pig farms and grants control over the annual number of pregnancies. Secondly, it enhances the productivity of farmers and optimizes equipment utilization. Lastly, it diminishes the yearly replacement rate of sows, thereby decrease the biosecurity risks of pig farms. Moreover, this model has been found to effectively decrease the NPD of sows and enhance their economic worth [1, 2]. Nevertheless, the drawbacks of this approach have become evident due to the occurrence of ASF outbreaks.

African swine fever virus (ASFV) is an enveloped DNA virus, which belongs to the *Asfivirus* genus within the *Asfarviridae* family [3]. The absence of commercially accessible vaccines or medications against ASFV presents growing difficulties for the swine industry. Our team has previously demonstrated the effectiveness of the “Whole-herd-Sampling, qPCR-based-Testing, and Precision-Removal method” for rapid virus clearance in ASFV-positive pig farms [4]. Nevertheless, this approach is not without its limitations, as it necessitates the complete cessation of production activities within the facility during the disposal process, such as mating operations. Consequently, this leads to an indirect economic loss as there will be no piglets born after a production cycle (114 days). To mitigate such indirect losses, this study selected several farms to conduct a proper Standard Operating Procedure (SOP) in the state of production halt during the disposal process.

## Case presentation

In this study, a total of twenty-four large sow farms, all of equal size, were chosen. Each farm consisted of two mating herds and ten farrowing herds. Each mating house accommodated 1200 sows, while each farrowing house housed 60 sows, each with an average litter size of 10 piglets.

**Fig. 1:**
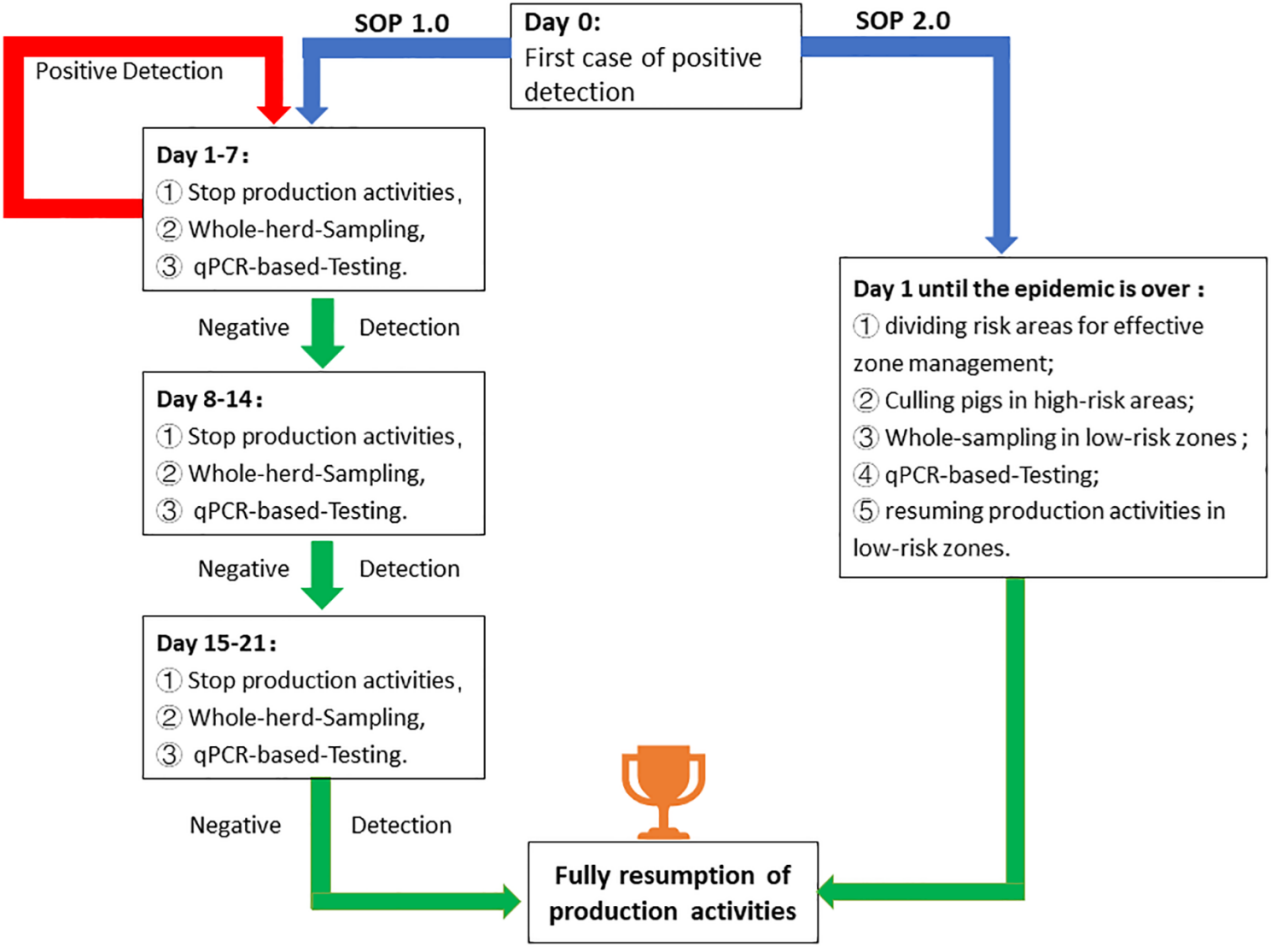
Flowcharts of SOP 1.0 and SOP 2.0

In previous approaches to managing outbreaks of ASFV, namely SOP 1.0 (Fig. 1), a facility-wide production cessation is enforced to contain the further transmission of the disease. Concurrently, a thorough detection and targeted eradication strategy is implemented, guided by the outcomes of qPCR testing conducted on the entire population. The incubation period of ASF typically spans from 3 to 19 days, with a maximum duration of 21 days [5, 6]. Consequently, the resumption of production activities can only occur following three consecutive rounds of comprehensive population testing, with each round comprising 7-day intervals. If an ASFV-positive sample is detected during this period, a new round of testing is initiated. In the context of practical production operations, we have identified an operational procedure, namely SOP2.0 (Fig. 1), which enables mating activities to be conducted during ASF outbreaks. This approach primarily entails the division of risk areas for effective zone management. The specific operational procedure is as followed. Firstly, upon discovering an ASFV-positive pig, immediate zone management is implemented for the pigsty. The high-risk zone includes the areas where the positive pig shares the same water trough and gutters with other pigs, as well as the areas managed by the same farmer in the past week. The remaining areas, sharing the same water trough and gutters, are defined as low-risk zones. Additionally, each zone consists of 120 pigs. Secondly, pigs within the high-risk zone are promptly isolated within 24 h following the outbreak, and their enclosures are treated with disinfectants, such as caustic soda. Thirdly, in low-risk zones, all pigs are subjected to tonsil swab testing, and if no positive results are detected, mating operations are recommenced. It is imperative to highlight that tonsil swab testing must be carried out on the entire pig population in low-risk zones prior to each mating operation. In contrast, the conventional procedure entails conducting a comprehensive herd testing on a weekly basis following an outbreak. Mating operations are only reinstated when the entire herd consistently yields negative results for a duration of three consecutive weeks.

Based on the aforementioned approaches, a comparative analysis was conducted on the prognosis outcomes of 24 herds that had encountered ASFV outbreaks. Out of these herds, 12 farms employed the SOP 1.0 method, while the remaining 12 farms implemented the SOP 2.0 method. The assessment primarily focused on several indicators, namely the days of epidemic disposal (DED), the overall count of positive detections, the retention rate, the NPD, the economic loss and the cost implications associated with the two approaches. The monetary value of RMB 5,500 per sow is determined based on the number of pigs present in these farms and the average price of pigs. Based on the number of pigs present in the farms and the average market value of pigs, specifically RMB 5,500 per sow and RMB 300 per piglet, the economic loss resulting from a 5% decrease in sow population can be calculated as 1200 × 2 × 5% × 5500, amounting to 660,000 RMB. Consequently, the formula for calculating the direct economic loss is expressed as follows: the economic loss (ten thousand yuan) = 66×N, where N equals (1 - the retention rate) divided by 5%. Additionally, considering the production rhythm of these farms, where 120 litters of piglets are slaughtered on a weekly basis, the economic loss incurred for each additional week of NPD can be determined as 120 × 10 × 300, equaling 360,000 RMB. Consequently, the formula for calculating the indirect economic loss is expressed as follows: the economic loss (ten thousand yuan) = 36×M, where M represents the NPD divided by 7 and multiplied by 36, with M being an integer.

Moreover, the evaluation of each approach also takes into account the associated expenses. As the disparity between SOP 1.0 and SOP 2.0 primarily lies in the methods used for disposing of mating herds, we solely consider the costs related to mating herds in this calculation. The expenses for disposal procedures mainly encompass the cost of sampling tools, labor expenses for sampling, testing fees for samples, and additional management costs. Sampling tools incur a cost of RMB 1 per pig. Each person’s labor cost for sampling amounts to RMB 2.5 per pig. The testing fee for each sample is RMB 6. Hence, the aggregate expenditure for sampling and testing per pig amounts to RMB 9.5. Within the SOP 1.0 disposal procedure, the primary expenses pertain to sampling and testing, encompassing a comprehensive herd examination on a weekly basis, while no supplementary costs are necessitated for management. Consequently, the formula for calculating the cost linked to SOP 1.0 for every farm is expressed as follows: the cost (ten thousand yuan) = 9.5 × 2400×WED (weeks of epidemic disposal) /10,000, wherein WED is derived from DED, and if its value is less than one week, it shall be considered as a week for calculation purposes.

Within the SOP 2.0 disposal procedure, the primary expenses are attributed to the costs associated with sampling, testing, and additional management. Firstly, it is necessary to determine the number of sows required for farrowing based on the mating week batches, which amounts to 120 sows. Assuming an average farrowing rate of 90%, the number of sows to be mated per week is calculated as 120/90%≈133, which are distributed across two zones. Consequently, a total of 240 pigs from the two zones must be sampled and tested on a weekly basis. Furthermore, the implementation of SOP 2.0 necessitates the allocation of additional labor resources to manage more independent zones to avoid cross-transmission of ASFV. Typically, the management of two mating herds necessitates the presence of eight individuals. However, in the context of SOP 2.0, these two herds are partitioned into twenty zones, thereby necessitating an additional twelve personnel. Each person is remunerated with a daily wage of RMB 200. Consequently, the formula for calculating the cost linked to SOP 2.0 for every farm is expressed as follows: the cost (ten thousand yuan) = (9.5 × 240×WED + 12 × 200×DED) /10,000.

All the data were subjected to statistical analysis using the unpaired t-test in the GraphPad Prism software (version 8.0).

**Fig. 2:**
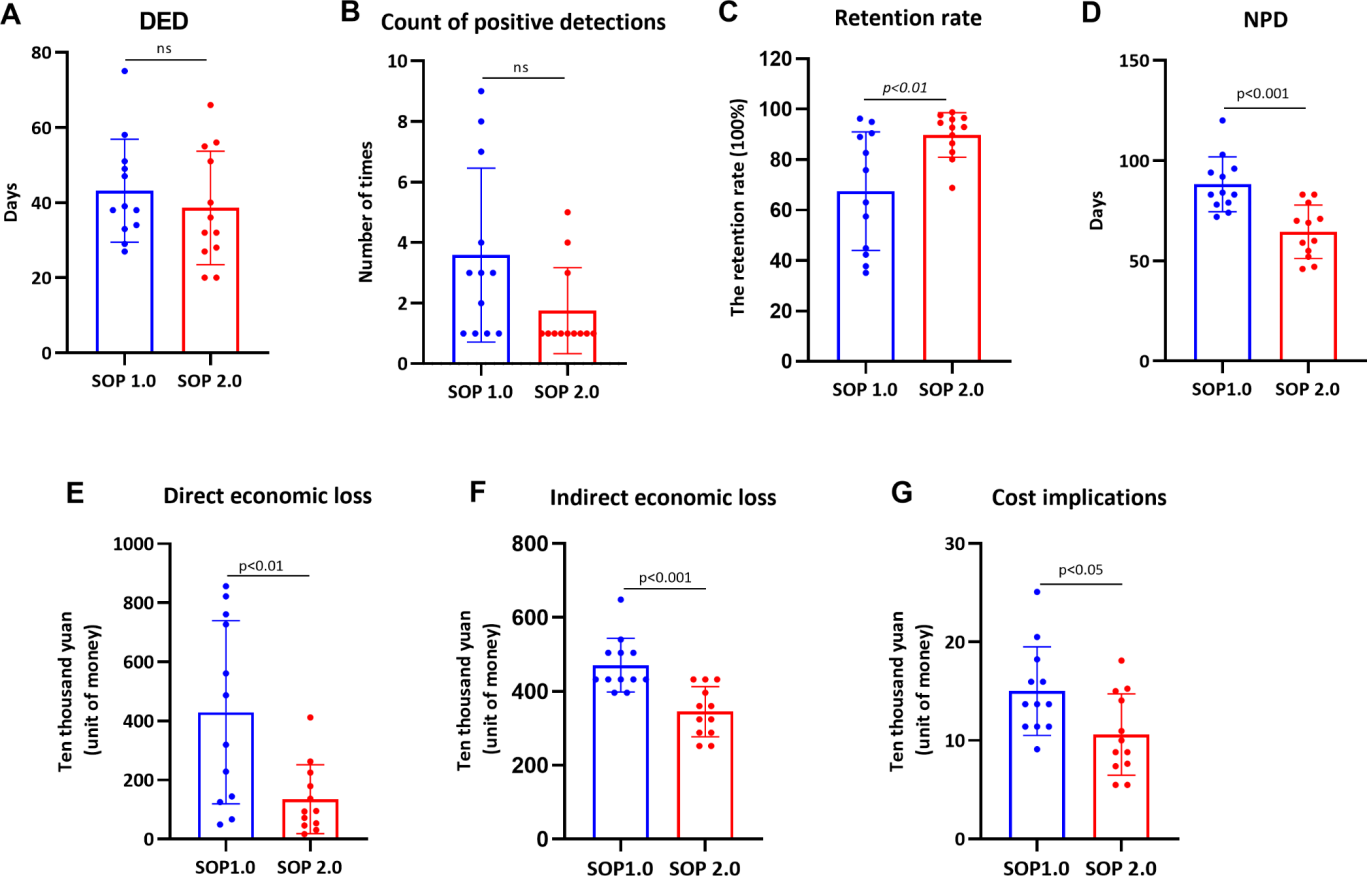
Comparative analysis of the prognosis results with two SOPs. **(A)** The DED, **(B)** the count of positive detections, **(C)** the retention rate, **(D)** the NPD, **(E)** the direct economic loss, **(F)** the indirect economic loss, and **(G)** the cost implications

The results revealed that during the epidemic period, as shown in Fig. 2A, the average DED in farms following SOP 1.0 was 43 days, while in farms implementing SOP 2.0, it was 38 days, indicating a 5-day reduction in disposal time compared to SOP 1.0 (p>0.05). Secondly, farms adhering to SOP 1.0 exhibited a lower number of positive detections compared to farms implementing SOP 2.0 (p>0.05), suggesting that the implementation of zone managements effectively mitigated the risk of cross-contamination and virus spread within the farms (Fig. 2B). Thirdly, the retention rate of pigs in farms implementing SOP 2.0 exhibited a significant increase compared to those adhering to SOP 1.0 (p<0.01) (Fig. 2C), aligning with the findings of positive detections. This observation further implies a decrease in direct economic losses incurred from the culling of pigs due to the epidemic (Fig. 2E). Fourthly, the outcomes of NPD analysis demonstrated that the implementation of SOP 2.0 significantly reduced the duration of non-productive days during the epidemic period (p<0.001) (Fig. 2D), facilitating the optimal utilization of sow production capacity and mitigating indirect economic losses associated with the epidemic. Fifthly, as shown in Fig. 2E F, the results of both direct and indirect economic losses showed that farms adhering to SOP 2.0 experienced significantly reduced economic losses compared to those following SOP 1.0 (p<0.01). Finally, as shown in Fig. 2G, the costs of SOP 2.0 were also significantly lower than those of SOP 1.0, indicating a better choice of SOP 2.0 for adoption. Above all, the rigorous implementation of SOP 2.0 enables uninterrupted production operations during an ASF outbreak, thereby effectively mitigating the substantial losses incurred by covert production and concurrently reducing the cost implications during disposal procedures.

## Discussion and conclusions

ASF has been detected in China for a duration exceeding five years, commencing in August 2018. Its rapid dissemination throughout the entire country has emerged as a significant peril to the domestic pig industry in China [5, 7]. In light of the various modes of transmission of ASF among pigs [8–10], our research team proposed the “Whole-herd-Sampling, qPCR-based-Testing, and Precision-Removal method” to address ASFV infection in large-scale pig farms [4]. The successful implementation of this technique has demonstrated the potential for remediation, even in instances where ASFV breaches biosecurity barriers and infiltrates farms. In the context of batch production models, outbreaks of ASFV can result in not only direct economic losses, such as the culling of pigs, but also indirect economic losses. These indirect losses include disruptions in production rhythms, decreased reproductive performance due to missed breeding periods, increased annual culling rates of sows, and reduced survival rates of piglets in farrowing herds [2, 11, 12]. It is worth noting that these indirect losses can even exceed the direct losses in terms of economic impact.

The implementation of this novel approach (SOP 2.0) has effectively eradicated the virus from pig populations across 12 farms, while concurrently ensuring uninterrupted production, thereby safeguarding normal production operations and controlling the epidemic. Adhering to the operational protocols outlined in SOP 2.0 and accurately demarcating and managing distinct zones during the epidemic period can substantially mitigate the adverse consequences of ASF outbreaks. The pivotal element of this procedure lies in segregating areas and meticulously designating breeding zones exclusively for safe production operations. The inclusion of zone management in the ASF prevention and control guidelines by the World Organisation for Animal Health (OIE) has been previously documented [13]. Furthermore, the application of SOP 2.0, while adding additional management costs of more independent zones, significantly reduces the total expenses by significantly reducing sampling and testing costs. This study adopts a regional division approach that takes into account the specific characteristics of ASFV transmission. The division of zones is determined by the transmission characteristics of ASFV, considering the highly contagious nature of the virus [14]. Consequently, shared water troughs and manure passages are treated as a single zone. Furthermore, the disinfection and inactivation of the virus in the environment are deemed equally crucial aspects to consider. The aerosol transmission of ASF has also gained attention among farmers [15, 16]. Consequently, it is imperative to consider future measures aimed at eradicating the virus present in aerosols. Furthermore, this study has presented a relationship model linking economic losses, retention rates, and NPD, thereby offering a valuable reference for assessing the economic losses of ASF.

In conclusion, the implementation of SOP 2.0 has demonstrated its efficacy in mitigating indirect economic losses resulting from ASF outbreaks and decreasing the expenses of disposal processes, while maintaining retention rates and disposition days unaffected. This approach has been successfully validated in pig farming operations and exhibits promising prospects for wider application.

## Data Availability

The datasets used and analyzed during the current study are available from the corresponding author on reasonable request.

## List of abbreviations

ASF: African swine fever
ASFV: African swine fever virus
NPD: non-productive days
SOP: Standard Operating Procedure
DED: Days of epidemic disposal
WED: Weeks of epidemic disposal
